# Qualitative Evaluation of a High-Resolution 3D Multi-Sequence Intracranial Vessel Wall Protocol at 3 Tesla MRI

**DOI:** 10.1371/journal.pone.0160781

**Published:** 2016-08-17

**Authors:** Nikki Dieleman, Wenjie Yang, Anja G. van der Kolk, Jill Abrigo, Ka Lok Lee, Winnie Chiu Wing Chu, Jaco J. M. Zwanenburg, Jeroen C. W. Siero, Ka Sing Wong, Jeroen Hendrikse, Fiona Xiang Yan Chen

**Affiliations:** 1 Department of Radiology, University Medical Center Utrecht, Utrecht, The Netherlands; 2 Department of Medicine, Chinese University of Hong Kong, Shatin, Hong Kong SAR, China; 3 Department of Imaging and Interventional Radiology, Chinese University of Hong Kong, Shatin, Hong Kong SAR, China; 4 Image Sciences Institute, University Medical Center Utrecht, Utrecht, The Netherlands; 5 Spinoza Centre for Neuroimaging, Amsterdam, The Netherlands; Heinrich-Heine-Universitat Dusseldorf, GERMANY

## Abstract

**Background and Purpose:**

Intracranial vessel wall imaging using MRI has great potential as a clinical method for assessing intracranial atherosclerosis. The purpose of the current study was to compare three 3T MRI vessel wall sequences with different contrast weightings (T_1_w, PD, T_2_w) and dedicated sagittal orientation perpendicular to the middle cerebral artery, to the reconstructed sagittal image from a transverse 3D T_1_w volumetric isotropically reconstructed turbo spin-echo acquisition (VIRTA), and provide a clinical recommendation.

**Materials and Methods:**

The above-mentioned sequences were acquired in 10 consecutive Chinese ischemic stroke or TIA patients (age: 68 years, sex: 4 females) with angiographic-confirmed MCA stenosis at 3T. Institutional review board approval was obtained. Two raters qualitatively scored all images on overall image quality, presence of artifacts, and visibility of plaques. Data were compared using Repeated measures ANOVA and Sidak’s adjusted post hoc tests.

**Results:**

All sequences except the T_2_w sequence were able to depict the walls of the large vessels of the Circle of Willis (p<0.05). T_1_w sagittal oblique VIRTA showed significantly more artifacts (p<0.01). Peripherally located plaques were sometimes missed on the sagittal sequences, but could be appreciated on the transverse T_1_w VIRTA.

**Conclusion:**

With the 3T multi-sequence vessel wall protocol we were able to assess the intracranial plaque with two different image contrast weightings. The sequence of preference to include in a clinical protocol would be the transverse 3D T_1_w VIRTA based on absence of artifacts, larger coverage including the whole Circle of Willis, and excellent lesion depiction.

## 1 Introduction

Intracranial atherosclerosis (ICAS) is a common cause of ischemic stroke worldwide, and the most important cause of stroke in the Chinese population, accounting for half of all strokes [1,2]. Patients with ICAS are also known to have a high recurrent stroke risk [3]. Lumenography-based methods like DSA and MRA are excellent to assess the (intracranial) vessel lumen. However, vasculopathies, including ICAS, affect the arterial vessel wall without luminal narrowing[4–8]. The pathological changes within the vessel wall lead to luminal narrowing only when the plaque is already in an advance stage[4–8], and may be missed by lumenography techniques. Therefore, in recent years, growing attention has been directed to imaging the intracranial arterial vessel wall. To avoid underestimation of wall pathologies, and thus disease severity, several intracranial vessel wall sequences have been developed in the past decade [8–11]. These sagittally oriented sequences were initially planned perpendicular to the vessel of interest, with relatively thick slices (>2mm) and a small FOV (covering only a few cm’s of the middle cerebral artery), but with a high in-plane spatial resolution [12]. The limited FOV was required to limit scan duration, and led to planning preferentially on a visibly stenotic area of the vessels, which is known to indicate the presence of advanced plaques. An alternative would be a transverse sequence that covers not only the stenosis, but also other main vessels of the circle of Willis. Less advanced plaques may be visualized even when luminal narrowing has not taken place, and scanning is less operator-dependent compared with sagittal sequences that need to be planned perpendicular to the stenosis.

Previous studies using a sequence with (larger) transverse coverage at 7T already demonstrated that most intracranial vessel wall lesions do not cause luminal stenosis [13,14]. These results indicate that a transverse vessel wall sequence would be a suitable tool to screen the Circle of Willis for intracranial vessel wall pathology. However, 7T MR systems are not widely available and not accepted as clinical standard. Therefore, 3T MRI would be more feasible to use in the clinical setting, but so far only a few studies [15,16] have used such sequences at 3T and none of these compared the transverse sequences to the more commonly used sagittal sequences that are planned on the side of the stenosis. Theoretically, because of the higher in-plane spatial resolution of the sagittally planned sequences, lesions would be more conspicuous compared with a sagittal reconstruction of a transverse sequence. However when the slice thickness of the transverse sequence is relatively small it might be that both sequences are equally good at depicting vessel wall lesions, even though the transverse sequence has a lower in-plane resolution, with the great advantage of covering all major branches of the Circle of Willis [13,15]. In the current study, we therefore compared sagittal vessel wall sequences with scanning axis perpendicular to the middle cerebral artery (MCA) with different contrast weightings (T_1_w, PD and T_2_w) to the reconstructed sagittal image of a transverse T_1_w vessel wall sequence, and provide a clinical recommendation.

## 2 Materials and Methods

### 2.1 Subjects

This prospective study was approved by the Institutional Review Board of the Chinese University of Hong Kong. All subjects gave written informed consent. Between February and September 2014, consecutive Chinese patients with a symptomatic MCA stenosis, as confirmed by conventional angiography, were included in this study. All patients had to be able to endure the MRI examination and had to have no contraindications for MRI. Patient characteristics including age, gender, and general vascular risk factors were collected during a patient’s visit to the hospital.

### 2.2 Imaging

MRI was performed on a 3T Achieva MR system (Philips Healthcare, Cleveland, OH, USA) with an 8-channel SENSE head coil. The protocol included a transverse 3D T_1_w volumetric isotropically reconstructed turbo spin-echo acquisition (VIRTA), a sagittal oblique 3D T_1_w VIRTA, a sagittal oblique 3D T_2_ -weighted T_2_w VIRTA, and a sagittal oblique 3D PDw VIRTA sequence. The protocol also included a TOF-MRA sequence. The scan parameters, including scan times, of all used sequences are given in Table 1. The transverse T_1_w VIRTA was adjusted from Qiao et al [15]. However, we used a repetition time of 1500ms and anti-DRIVen Equilibrium (DRIVE) module for increased T_1_-weighting and CSF suppression, respectively. Additionally, we used a minimum flip angle of 25° in the variable flip angle refocusing pulse train for increased flow suppression (in CSF and blood). [17] As a straightforward implementation of the sequence based on the parameters from the paper led to a scan time of 16 minutes (where Qiao reported a scan time of 7.9 minutes), we used a factor of 2 interpolation by zero-padding in the slice direction during reconstruction, and further reduced the scan time by slightly adjusting the acquired in-plane resolution and reducing the TR. The resulting sequence, with a reconstructed isotropic voxel size of 0.5 mm^3^, had a scan time of 6:51 minutes.

**Table 1:** Scan parameters of the acquired 3D sequences.

	T_1_w VIRTA transverse	T_1_w VIRTA sagittal	T_2_w sagittal sequence	PDw sagittal sequence
**Field-of-view (mm**^**3**^**)**	200x167x45	200x167x35	200x167x35	200x167x35
**Acquired resolution (mm**^**3**^**)**	0.6x0.6x1.0	0.6x0.6x1.0	0.7x0.7x1.0	0.6x0.6x1.0
**Reconstructed resolution (mm**^**3**^**)**	0.5x0.5x0.5	0.5x0.5x0.5	0.6x0.6x0.5	0.3x0.3x0.5
**Slice orientation**	Transverse	Sagittal	Sagittal	Sagittal
**TR/TE (ms)**	1500/36	1500/36	2500/384	1300/31
**SENSE**	1.5	1.5	2	2.2
**Echo spacing**	4.0	4.1	3.8	6.1
**TSE + startup echoes**	56 + 6	56 + 6	133 + 6	65 + 4
**Oversampling factor**	1.8	1.8	1.8	1.8
**Scan duration (min)**	06:51	05:52	03:37	04:07

For 6 patients we also obtained post-contrast scans. Before acquisition of the contrast enhanced T_1_w VIRTA sequence, 0.1 mL/kg of a gadolinium-containing contrast agent (Dotarem, Gadoteric acid 0.5 mmol/mL, Guerbet, Roissy CdG Cedex, France) was administered to the patient.

### 2.3 Image analysis

Precontrast images were processed on a workstation (Philips) and with MeVisLab v2.5 (MeVis Medical Solutions AG, Bremen, Germany). A sagittal reconstruction was made of the transverse T_1_w VIRTA sequence; in this way care was taken that all images were in the sagittal orientation. Qualitative image analysis was performed independently by two raters (ND: 3 years of experience and AK: 6 years of experience in scoring vessel wall images). Raters were blinded for clinical information. Analyses were performed according to the methods described by Van der Kolk et al [18]. First, a side-by-side analysis [19] was performed, in which raters were individually presented with random pairs of unannotated images (apart from their experience with the contrast), which were scored based on overall image quality of the Circle of Willis using a three-point scale (worse; equal; better). The analysis serves as a paired test, where the raters were asked to choose one of the images as superior, and choose equal when no differences were apparent. [18,19] Overall percentages of the cases in which one technique was found to be better, equal or worse were calculated. A sequence was considered superior if the overall image quality score was better in more than 75% of the cases. Second, images were randomly shown to the raters and three qualitative evaluations were performed as follows: image quality (poor; adequate; good), presence of artifacts (not present; moderate; present with influence on diagnosis), and visibility of the plaque (poor; adequate; good). Scores are presented as median values.

### 2.4 Statistical analysis

IBM SPSS version 20.0 for Windows was used for statistical analysis. Proportions of raw agreement were calculated to evaluate interrater agreement. Data were compared by using Repeated measures ANOVA; Sidak’s adjusted post hoc tests were performed for pairwise comparisons. Statistical significance was set at p<0.05.

## 3 Results

### 3.1 Subjects

**Fig 1:**
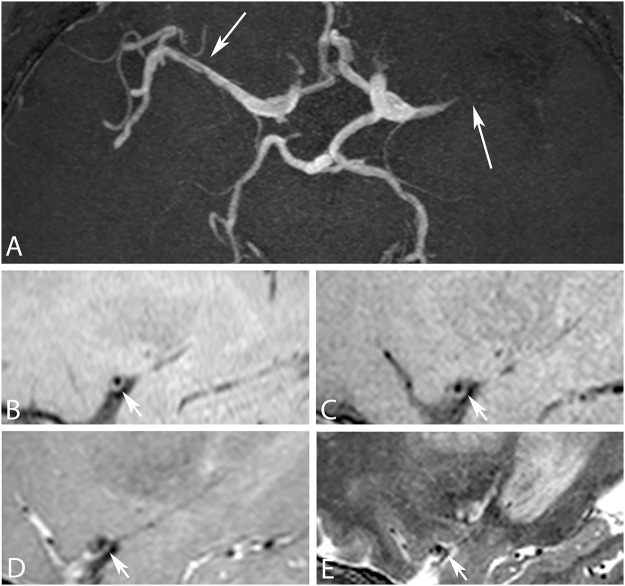
Comparison between four different 3D intracranial vessel wall sequences at 3 tesla in a 47-year-old male patient presented with a bilateral partial anterior circulation infarct as caused by an occlusion and stenosis in the left and right middle cerebral artery (MCA), respectively. (A) On the transverse 3D time-of-flight magnetic resonance angiography two stenoses can be appreciated, one in the left M1 segment of the MCA and one in right M1 segment of the MCA (arrows). (B) Sagittal reconstruction of the transverse T_1_w VIRTA, (C) sagittal T_1_w VIRTA, (D) sagittal PDw and (E) sagittal T_2_w images showing a vessel wall lesion in the left middle cerebral artery (arrows). On both T_1_w VIRTA images and the PDw image the plaque can be clearly delineated, however on the T_2_w image the vessel wall is less clear.

Between February and September 2014, ten patients (4 females; mean age 68 years, range 47–78 years) with symptomatic MCA atherosclerosis (total 11 stenoses) were studied at 3T MRI using an imaging protocol that included the above mentioned intracranial vessel wall sequences. Of these ten patients, 2 had had a transient ischemic attack and 8 had had an ischemic stroke (Table 2). No major (motion) artifacts that hampered image analysis were found. Example slices of the four sequences within one patient are shown in Fig 1.

**Table 2:** Patient demographics.

Patient ID	Side of stenosis	DSA stenosis (%)	Infarct location	TOAST diagnosis
1	Left	89	Left partial anterior circulation infarct	Large-artery atherosclerosis
2	Left	71	Left corona radiata and Internal capsule	Large-artery atherosclerosis
3	Left	92	Left lentiform nucleus and corona radiata	Large-artery atherosclerosis
4	Left	n.g.	Left lacunar infarct	Large-artery atherosclerosis
5	Left	70	none (TIA)	Large-artery atherosclerosis
6	Right	72	none (TIA)	Large-artery atherosclerosis
7	Left	n.g.	Left MCA internal borderzone, basal ganglia	Large-artery atherosclerosis
8	Left and Right	70	Right basal ganglia	Large-artery atherosclerosis
9	Left	80	Left parietotemporal lobe	Large-artery atherosclerosis
10	Left	75	n.g.	Large-artery atherosclerosis

n.g. = not given; MCA = middle cerebral artery; TIA = transient ischemic attack; TOAST = Trial of Org 10172 in Acute Stroke Treatment classification system

### 3.2 Image analysis

**Fig 2:**
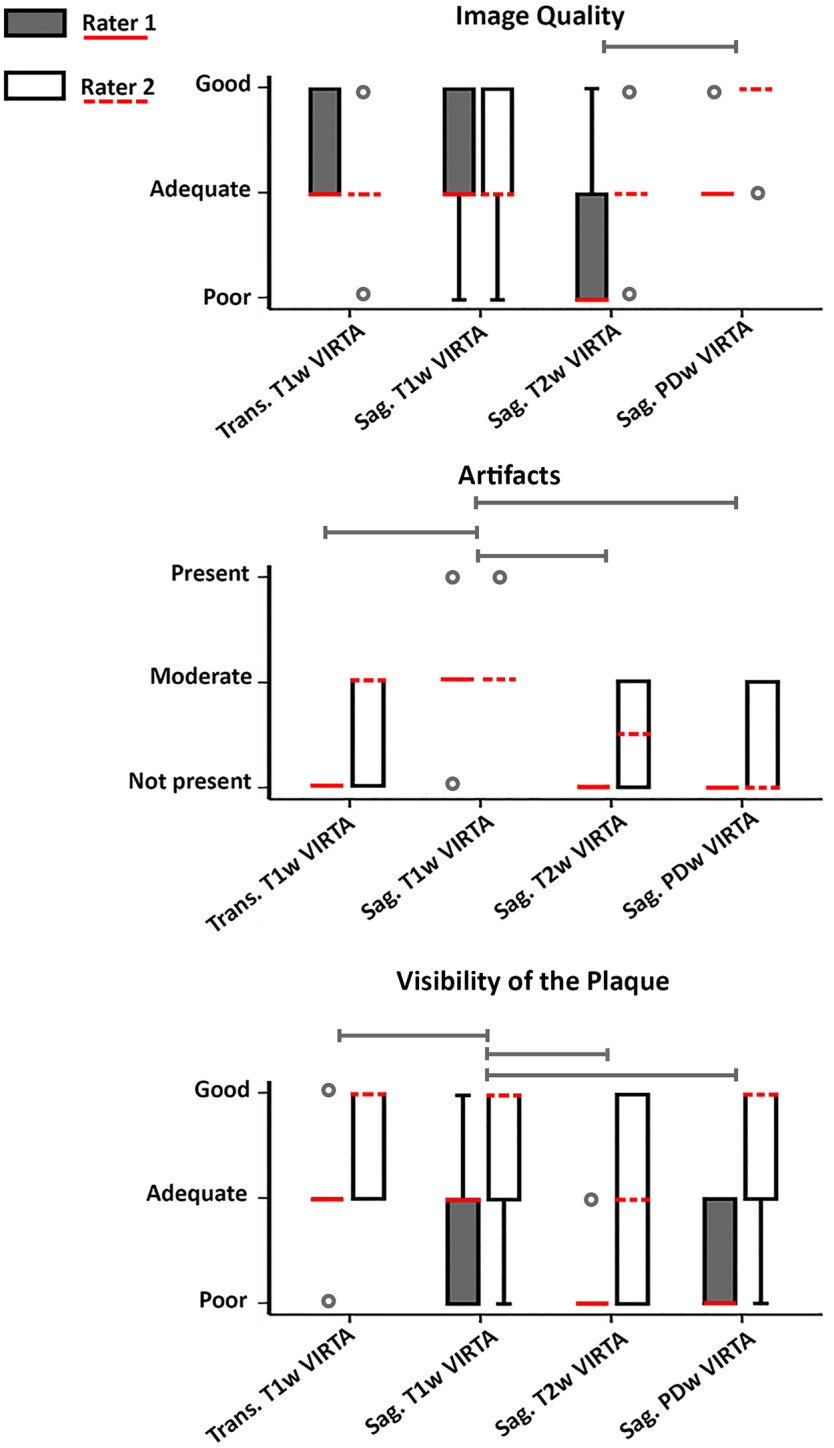
Box plots of image quality, artifacts, and visibility of the plaque for the two raters. Red line = median value of rater 1, dashed red line = median value of rater 2, box = interquartile range, whiskers = minimum and maximum values, and rounds = outliers. The image quality and visibility of the plaque were scored on a scale from 0 (poor) to 2 (good); the presence of artifacts was scored on a scale from 0 (not present) to 2 (present with influence on diagnosis). Some whiskers are not visible because the lower quartile is equal to the minimum, or the upper quartile is equal to the maximum. Also, some median values overlap with the upper or lower quartile. The median value of the artifacts of the T_2_w VIRTA is 0.5 because equal numbers are scored 0 and 1. Caps over the boxes represent significant differences, p-values for image quality, artifacts and visibility of the plaque were <0.001; <0.01; and <0.05, respectively, as assessed by repeated measures ANOVA and Sidak’s adjusted post hoc tests. As an example for the caps: in the upper panel there is an overall significant difference between the T_2_w VIRTA and the PDw VIRTA (p<0.001).

**Fig 3:**
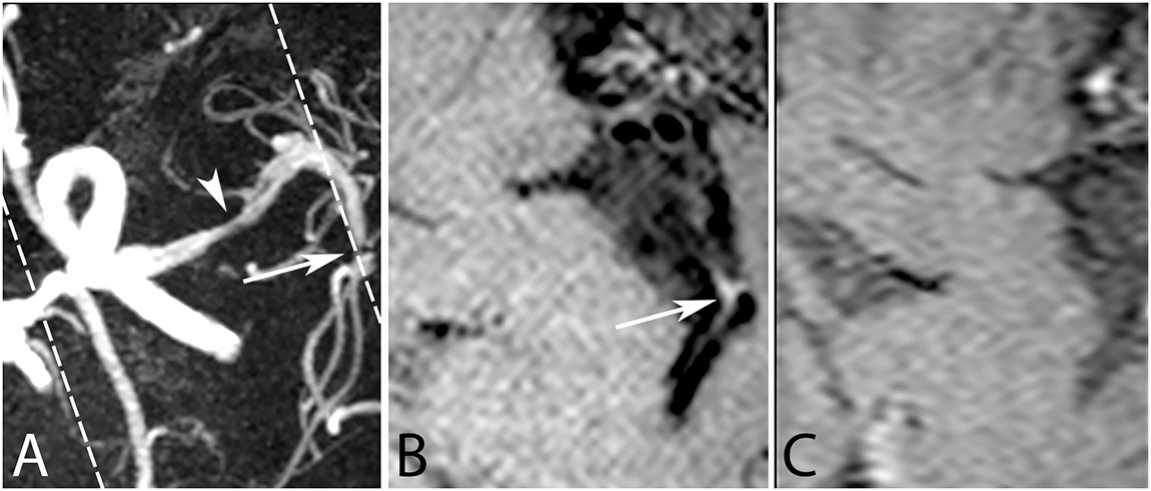
A 73-year-old female patient presented with subacute infarcts of the left parietal cortex and left internal borderzone area. (A) On the transverse 3D time-of-flight magnetic resonance angiography two stenoses can be appreciated, one in the left M1 segment of the middle cerebral artery (arrowhead) and one in M2 segment of the middle cerebral artery (arrow). The transverse T_1_w VIRTA, after contrast administration, shows a corresponding vessel wall lesion in the M2 segment (B), this lesion is however missed by the transverse reconstruction of the sagittal T_1_w VIRTA sequence, also after contrast administration (C) because of its limited field-of-view (indicated by the dashed lines in A).

Results of the qualitative assessment are reported in Table 3 and Fig 2. For the side-by-side comparison all four sequences had similar ratings, except the T_2_w sequence which was judged to give the worst overall visualization of the vessel wall in 60–80% of the cases (Table 3). Interrater agreement is given for all scores in Table 4. Individual scoring of the sequences is shown in Fig 2. Results in Fig 2 are presented as Box-and-Whisker plots given for both raters and all four methods (scale from 0 = poor to 2 = good, and for artifacts: 0 = not present and 2 = present and influencing diagnosis). Most important findings of these plots were that all sequences except the T_2_w sequence were able to sufficiently depict the vessel walls of the large vessels of the Circle of Willis (Fig 2, upper panel). Comparisons of the three qualitative analyses demonstrated that the PDw sequence provided better image quality than the T_2_w sequence (p<0.001), but showed equal image quality of the vessel wall when compared with the two T_1_w VIRTA sequences. Second, in the sagittal oblique T_1_w VIRTA images, artifacts were observed that were not observed within the same slice of the other sequences (p<0.01) (Fig 2, middle panel). Third, the plaque was clearly delineated from its surrounding in the T_1_w VIRTA sequences and the PDw sequence, but not in the T_2_w sequence (p<0.05) (Fig 2, lower panel and Fig 1). Furthermore, more peripherally located plaques found, for instance, in the M2 segment of the middle cerebral artery, were sometimes missed on the sagittal sequences, but could be appreciated on the transverse T_1_w VIRTA (Fig 3).

**Table 3:** Side-by-side comparison analysis of three different vessel wall sequences, relative to the sagitally reconstructed transverse T_1_w VIRTA.

Comparison (in percentages*)		T_1_w VIRTA sagittal	T_2_w sagittalsequence	PDw sagittalsequence
T_1_w VIRTA transverse	>	45	80	45
T_1_w VIRTA transverse	=	25	15	10
T_1_w VIRTA transverse	<	30	5	45
T_1_w VIRTA sagittal	>	-	75	50
T_1_w VIRTA sagittal	=	-	15	20
T_1_w VIRTA sagittal	<	-	10	30
T_2_w sequence	>	-	-	5
T_2_w sequence	=	-	-	35
T_2_w sequence	<	-	-	60

>, better;

=, equal;

<, worse;

* For each side-by-side comparison, 20 scores were obtained (10 cases x 2 observers), and the percentage (rounded) better than, equal to or worse was calculated per comparison.

**Table 4:** Proportions of raw agreement values for the four different sequences and three different measurements.

	Proportions of agreement		
	Image quality	Artifacts	Visibility of plaque
Transverse T_1_w VIRTA	0.6	0.3	0.6
Sagittal T_1_w VIRTA	0.6	0.7	0.5
Sagittal T_2_w VIRTA	0.4	0.5	0.5
Sagittal PDw VIRTA	0.3	0.6	0

## 4 Discussion

In this study we have compared four different intracranial vessel wall sequences. We showed that intracranial atherosclerotic plaques were clearly visible with good image quality in ten patients with a symptomatic MCA stenosis, using a sagittal oblique T_1_w VIRTA sequence, a sagittal reconstruction of a transverse T_1_w VIRTA sequence and a PDw sequence. The T_2_w VIRTA sequence was not of sufficient quality to be clinically usable. The transverse scan had the advantage of imaging both hemispheres at once; covering the total Circle of Willis instead of only a few cm’s of one of the MCA’s. Since the transverse T_1_w VIRTA sequence scored at least comparable regarding artifacts and lesion depiction relative to the sagittal scan with limited coverage, and had the advantage of whole Circle of Willis coverage, the transverse T_1_w VIRTA sequence was judged the best intracranial vessel wall sequence.

The transverse T_1_w VIRTA sequence was adjusted from Qiao et al., [15]. As mentioned above we used the anti-DRIVE module for better CSF suppression and a TR of 1500 ms for increased T_1_-weighting. From empirical data (unpublished), we know that the anti-DRIVE results in a better depiction of the vessel wall from its surrounding because of improved CSF suppression. The shorter TR (compared to Qiao et al.) increases the T_1_ weighting, thereby sensitizing the sequence for potential contrast enhancement of vessel wall lesions after contrast administration. Furthermore, in order to obtain a reasonable scan time (of approx. 7 minutes) we reduced the acquired voxel size to 0.6 x 0.6 x 1.0 m^3^. Therefore, the acquired resolution was not isotropic, but the reconstructed resolution is. However, the sagittal reconstruction of the transverse sequence was found to be equally sufficient to depict the vessel wall as the true oblique sagittal sequences. The clear advantage of the faster acquisition is that more patients can tolerate the scan and the sequence (6:51min) can be implemented more easily in a clinical protocol.

Although the PDw and the sagittal T_1_w VIRTA sequences provided good image quality in the qualitative analysis, comparable to the reconstructed sagittal images from the transverse T_1_w VIRTA sequence, this latter sequence is in our opinion the best sequence to use in clinical practice. It can cover the entire Circle of Willis at once, and can be reconstructed in any direction allowing for equal views as obtained by the sagittally oriented sequence. This delivers the MR technicians from the need of precise planning perpendicular to the wall, aiming for a (preferentially stenosed) part of a single branch only, as is common practice for the sagittal sequence, due to the limited FOV in the slice encoding direction, and increases the robustness of use in daily clinical practice by MR technicians with planning of the T_1_w VIRTA similar to the TOF MRA centered around the branching vessels of the Circle of Willis.

Although there were differences between the two raters for the qualitative image analysis, it can be appreciated that they did not contradict each other, since there seems to be a systematic bias in judgment of the sequences and image analyses. For example, the second rater judged visibility of the plaques systematically higher than the first rater, but the relative scores between the sequences was comparable (lowest boxplot in Fig 2). Nevertheless, the rating system we used was a subjective measurement. A rating system in which quantitative measures are used like SNR and contrast-to-noise ratio measurements within a region of interest may be of help to objectively judge sequences. However, for clinical purposes, the used qualitative analysis is maybe more representative for the daily practice.

In the current study, sequences with different contrast weightings were acquired and tested. From carotid vessel wall imaging it is known that these different image contrast weightings can aid in atherosclerotic plaque characterization [20–22]. Whether this also applies for intracranial atherosclerosis is not yet known. We do know that plaque enhancement, by means of contrast agent uptake, might be a marker for plaque vulnerability possible related to vasa vasora in the plaque and may reflect plaque rupture [23,24]. Moreover, intraplaque hemorrhage was demonstrated with the use of T_1_w imaging and PDw imaging [25,26]. Using multiple image contrast weightings may provide even more information about the vulnerability of a plaque by visualizing the presence of calcifications, fibrous cap, lipid-rich necrotic core, vasa vasorum and intraplaque hemorrhage. Ultimately, it may help us to better understand the pathophysiology of intracranial atherosclerosis and its role in ischemic stroke. With the presented transverse T_1_w-VIRTA sequence, we can develop other contrast weightings with a transverse orientation as well to be used in the clinic.

This study has several limitations. First, for the presented sequences, there is a tradeoff between the high spatial resolution needed to visualize the small intracranial arterial wall and a clinically reasonable scan time. Therefore, CSF is not completely suppressed and coverage of our sequences is still limited to the Circle of Willis or, in case of a sagittal sequence, one hemisphere. Using the sagittal sequences, planned perpendicular to the stenosis, plaques located in the other hemisphere may have been missed. Second, the acquisition time is still long (6:51min), which requires cooperative patients. However, in our experience, patients can handle the scan time quite well. Third, our sample size is relatively small, since our sequences were added as pilot to an already existing study. Furthermore because patients were recruited via different ways, not all received a contrast agent but analyses were performed on the precontrast scans only. Fourth, the contrast weightings of the intracranial vessel wall sequences are less strong than the weightings that are normally used for anatomical sequences and the voxel size of the T_2_w sequence was slightly lower as compared to the other sequences. This was done to maintain sufficient SNR. Hence, contrast between gray and white matter is less than what one maybe used to, especially in the T_1_w VIRTA sequence. However, from empirical data we know that the T_1_-weighting is strong enough for contrast enhancement, which is an important feature for plaque imaging. Fifth, at this moment only two *ex-vivo* studies have sought to validate MRI with histology [27,28], therefore, it is still not clear which lesions visualized at 3T are pathologic and which lesions are not (e.g., normal aging). Furthermore, we do not know what components, as seen on the MR images, we have identified. More validation studies should be performed in the future, ideally comparing *in-vivo* results with post-mortem histology, to validate our results.

### Conclusion

With the presented 3T multi-sequence vessel wall protocol we compared intracranial plaque imaging with different image contrast weightings. From these results, the sequence of preference to be included in a clinical protocol would be the transverse 3D T_1_w VIRTA based on overall absence of artifacts, excellent depiction of atherosclerotic plaque, and its ability to image the more generalized intracranial atherosclerotic burden.

## Abbreviations

DRIVE: anti-DRIVen Equilibrium
ICAS: intracranial atherosclerosis
MCA: middle cerebral artery
VIRTA: volumetric isotropically reconstructed turbo spin-echo acquisition
